# Protective role of salt in catalysis and maintaining structure of halophilic proteins against denaturation

**DOI:** 10.3389/fmicb.2014.00165

**Published:** 2014-04-09

**Authors:** Rajeshwari Sinha, Sunil K. Khare

**Affiliations:** Department of Chemistry, Indian Institute of Technology DelhiDelhi, India

**Keywords:** halophiles, haloadaptations, structure, denaturants, secondary structure, tertiary structure

## Abstract

Search for new industrial enzymes having novel properties continues to be a desirable pursuit in enzyme research. The halophilic organisms inhabiting under saline/ hypersaline conditions are considered as promising source of useful enzymes. Their enzymes are structurally adapted to perform efficient catalysis under saline environment wherein n0n-halophilic enzymes often lose their structure and activity. Haloenzymes have been documented to be polyextremophilic and withstand high temperature, pH, organic solvents, and chaotropic agents. However, this stability is modulated by salt. Although vast amount of information have been generated on salt mediated protection and structure function relationship in halophilic proteins, their clear understanding and correct perspective still remain incoherent. Furthermore, understanding their protein architecture may give better clue for engineering stable enzymes which can withstand harsh industrial conditions. The article encompasses the current level of understanding about haloadaptations and analyzes structural basis of their enzyme stability against classical denaturants.

## Introduction

Halophiles are the class of extremophiles which inhabit saline/hypersaline habitats. Halophilic proteins retain their structural and functional integrity under such high salt conditions (Oren, 2008). Certain unique structural features enable them to sustain their structure and physiological activities at high salt. These proteins, thus offer a unique model system to decipher structure function modulation under saline environment.

A perfect model that accurately explains how salts stabilize a protein is still debatable. Earlier studies on some of the extreme halophilic and haloarchaeal enzymes like *Haloarcula marismortui* malate dehydrogenases (Hm MDH) (Mevarech et al., 1977) and *Halobacterium salinarum* ferredoxins (Gafni and Werber, 1979) indicated that their enzymatic properties are fully expressed only in the presence of salt and that the gradual withdrawal of salt leads to the unfolding of protein. However, current understanding has emerged that requirements of high salt for activity and stability would rather be a restrictive definition of the halophilic proteins isolated from extreme halophiles. In many cases such as MDH from *Salinibacter ruber* (Madern and Zaccai, 2004), α-amylase from *Har. hispanica* (Hutcheon et al., 2005), the enzyme is not completely inactivated in the absence of salt. Hm MDH from different studies have shown the enzyme to be stable at millimolar concentration of salts in the presence of divalent cations or coenzyme (Bonneté et al., 1994; Madern and Zaccai, 1997).

Haloadaptations of proteins *viz*. presence of increased number of acidic amino acid residues on protein surface, smaller hydrophobic patches as well as salt bridges between acidic and strategically positioned basic residues have been previously defined (Lanyi, 1974; Eisenberg et al., 1992; Danson and Hough, 1997; Madern et al., 2000). Since then, structural analyses have revealed two significant differences in the characteristics of the surface of the halophilic enzymes. The first of these is that the excess of acidic residues are predominantly located on the enzyme surface forming a hydration shell that protects the enzyme from aggregation under high salinity. Secondly, the surface also displays a significant reduction in exposed hydrophobic character, which arises from a reduction in surface-exposed lysine residues. Oren (2013) recently reviewed the occurrence of acidic proteomes in halophiles for a better understanding of the modes of haloadaptation at the cellular level.

The halophilic proteins remain highly soluble in high salt milieu whereas their non-halophilic counterparts precipitate. The most appropriate model that explains the changes in solvent properties of halophilic proteins is the “solvation-stabilization model” (Ebel et al., 1999; Costenaro et al., 2002; Ebel et al., 2002). The thermodynamic basis in such cases has been well explained by Zaccai (2013). The model reflects that solubility, and changes in stability are intrinsically coupled. Extensive characterization of orthologous enzymes from extreme halophilic microorganisms, have shown that the unique adaptive feature which is shared by stable and unstable halophilic proteins is their high solubility at high salt concentration (Coquelle et al., 2010). Crystallographic analysis on halophilic and non-halophilic MDH from *Salinibacter ruber* and *Chloroflexus aurantiacus* respectively successfully established that acidic amino acids in the former were involved in disruption of pentagonally arranged network of water molecules within the hydration shell of halophilic protein (Talon et al., 2014). The acidic enrichment was attributed to an “evolutionary innovation” which enabled the protein to adapt under saline stress and suitably alter inherent protein-solvent interactions. A correlation between an increase of acidic amino acid and a favorable change of solubility in halophilic proteins has also been established by Tadeo et al. (2009).

A good amount of data has emerged in recent years which indicate a protective role of salt in stabilizing these proteins against classical denaturants. The present article encompasses the major haloadaptations directed toward understanding the basis of stability against classical denaturants. A critical understanding of how halophilic proteins, under the influence of salt, retain structural and functional integrity amidst denaturing milieu will provide guidelines and templates for engineering stable proteins/enzymes for industrial applications.

## Basic aspects in protein stability

The effects of salt on structure and function of non-halophilic proteins have been well worked out. The presence of high salt in a protein solution will have the following implications: disturbance in local water structure around the protein; decreased propensity for intermolecular hydrogen bonds, affecting protein solubility, binding, stability and crystallization; increased surface tension of water, striping off the essential water layer from the protein surface and increased hydrophobic interactions, causing protein aggregation and precipitation. To sum up, the protein structure and consequent functions are adversely affected by high salt concentrations.

## Protein denaturation

Denaturation of a protein refers to the loss of biological activity due to structural changes in the protein brought about by physical or chemical factors such as pH, temperature, salt, detergents, organic solvents or chaotropic agents. The secondary, tertiary or quaternary structures are largely affected upon denaturation. Some of the important mechanisms of protein denaturation are (http://class.fst.ohio-state.edu/FST822/lectures/Denat.htm):

- High temperature weakens the inherent bonds in protein. Further heating disrupts the hydrogen bonds within protein and new hydrogen bonds are established with surrounding water molecules thus breaking the helical structure.
- Water miscible solvent (less polar than water) lowers the dielectric constant of the system thereby strengthening electrostatic interactions among molecules. The protein largely unfolds exposing hydrophobic groups to the solvent causing aggregation and precipitation.
- Proteins are usually more soluble in dilute salt solutions because the salts in their ionic forms associate with opposite charges within the protein moiety, leading to increased hydration of the surface. However, at very high salt concentration, the increased surface tension of water generates a competition between protein and salt ions for hydration. Salts strip off the essential layer of water molecules from the protein surface eventually denaturing the protein.
- Protein denaturation by urea may occur by direct or indirect mechanisms (Lindgren and Westlund, 2010). Urea may directly interact with proteins by hydrogen bonding with the polarized areas on protein surface, weakening intermolecular bonds and protein structure. Guanidium hydrochloride (GdmCl) has a similar mechanism but is a more effective denaturant than urea. In an indirect mechanism, urea may disturb the water structure causing destabilization of the protein.
- Acids and bases alter the pH of the solution as well as disrupt the salt bridges which are primarily stabilizing ionic interactions between opposite charged amino acid residues on protein surface. Heavy metal salts *viz.* Hg^2+^, Cd^2+^, Pb^2+^, Ag^+^ similarly disrupt salt bridges or disulfide linkages in proteins leading to an insoluble metal protein complex. SDS induced protein denaturation involved unfolding of tertiary structure and “chain expansion” (Bhuyan, 2010).

## Salt is essential for maintaining structure and function of halophilic proteins

Evidently, presence of salt is a prerequisite for functioning of halophilic proteins (Mevarech et al., 2000). Large number of studies have been undertaken to investigate the role of salt in regulating structures and function of extremely/ moderately halophilic enzymes. Some of these are summarized in Table 1. The data suggests at the precise salt dependence of protein structures in halophiles. Majority of the studies indicate loss of enzymatic activity upon salt removal. These observations have been well supported by structural data. While the protein remained predominantly unfolded or randomly coiled in salt free medium, salt promoted increase in negative ellipticity and subsequent refolding. The effect of other metal ions on the activity and stability of halophilic enzymes have also been investigated. Differential roles of divalent Ca^2+^ and monovalent Na^+^ in preventing unfolding and regulation of catalytic activity respectively was also reported recently for *Bacillus* sp. EMB9 protease (Sinha and Khare, 2013a). Salt and divalent metal ions were reported to independently stabilize and regulate catalysis and folding of RNase H1 from *Hbt.* sp. NRC-1 (Tannous et al., 2012). In a different study, changes in tertiary structure of ferredoxin were associated with removal of Fe^3+^ (Gafni and Werber, 1979).

**Table 1:** **Effect of salt on activity and structure of halophilic enzymes**.

**Enzyme**	**Microorganism**	**Salt range investigated**	**Structure-function correlation**	**Reference**
Malate dehydrogenase	extreme halophilic bacteria from the Dead Sea	0–4.0 M NaCl	Salt withdrawal caused loss of enzymatic activity Distorted ellipticity with complete loss of secondary structure	Pundak and Eisenberg, 1981
NADP-glutamate dehydrogenase	*Haloferax mediterranei*	33 mM	Fluorescence spectra remained unaltered until KCl concentration was lowered to 33 mM At 33 mM KCl, time dependent decay of activity and complete fluorescence quenching	Ferrer et al., 1998
Thermolysin	*Bacillus thermoproteolyticus*	1–5 M NaCl	Activity enhanced by 15 fold in presence of 4 M NaCl Heat stability doubled in presence of 1.5 M NaCl supported by CD spectral data	Inouye et al., 1998
Transducers HtrX and HtrX1	*Halobacterium salinarum*	0.2–3.6 M NaCl or KCl	Under low-ionic-strength conditions (~0.2 M NaCl or KCl) HtrXI assumed a random coil structure while HtrX retained 85% α-helicity Addition of NaCl or KCl led to increase in α-helical characters for both	Larsen et al., 1999
Fe2-S2 ferredoxin	*Halobacterium salinarum*	0.1–4.5 M	Unfolding at low salt; time dependent loss of secondary structure Destabilization of the Fe2-S2 center of ferredoxin at low salt	Bandyopadhyay and Sonawat, 2000
Dihydrofolate reductase (DHFR)	*Haloferax volcanii*	0.12 M and 3 M KCl	*Hv* DHFR inactivation below 0.5 M KCl Secondary and tertiary structures showed analogy with the activity trend	Wright et al., 2002
Nucleoside diphosphate kinase (NDK)	*Natrialba magadii*	90 mM–3.5 M NaCl	Red shift of λ_max_ from 333 nm (at 1.75 M NaCl) to 340 nm (without NaCl) in fluorescence spectra	Polosina et al., 2002
Isocitrate dehydrogenase (ICDH)	*Haloferax volcanii*	0–5.0 M NaCl	Dissociation of the protein at low NaCl High α-helical content at 4 M NaCl was indicative of a fully folded active enzyme while partially folding observed at low concentration Irreversible denaturation below a threshold concentration	Madern et al., 2004
α-amylase AmyH	halophilic archaeon *Haloarcula hispanica*	0–4.0 M NaCl	Retained structural and functional integrity in the absence of salt Intrinsic fluorescence suggest that it did not unfold at low salt but may get slightly more loosely folded Very stable in high-salt buffer	Hutcheon et al., 2005
Esterolytic enzyme LipC	archaeon *Haloarcula marismortui* and overexpressed in *E. coli* BL21	0–5.1 M NaCl	Far UV-CD showed maximum negative ellipticity at 3.4 M NaCl. Considerable loss of secondary structure, as salt concentration was varied away from the optimal value	Rao et al., 2009
Recombinant esterase	*Haloarcula marismortui*, (Hm EST), cloned and overexpressed in *E. coli*	0–500 mM KCl	Unfolded protein in salt-free medium Pronounced negative ellipticity, increase in α-helical content upon addition of KCl	Müller-Santos et al., 2009
Protease	*Geomicrobium* sp. EMB2	0–10% (w/v) NaCl	Secondary structure of the protease unfolded in salt-free medium Structure regained by inclusion of 2–5% NaCl	Karan and Khare, 2011
Nep extracellular protease	*Natrialba magadii*	1.0–3.0 M NaCl	Irreversible denaturation, aggregation and loss of activity of Nep in the absence of salt; random coil structure in CD spectra Secondary conformation sufficiently folded in salinity profile	Souza et al., 2012

However, anomalies to the above generic trend cannot be ruled out. *S. ruber* MDH and *Har. hispanica* amylase remained completely active and structured even in absence of salt (Madern and Zaccai, 2004; Hutcheon et al., 2005). Likewise, glutamate dehydrogenase (GDH) from *Hbt. salinarum* was catalytically active under both low and high salt (Ishibashi et al., 2002). The exact reason for such stability is not known but it is plausible that the folded protein is structurally rigid enough to remain folded in the correct conformation and withstand non-saline environment.

## Effect of denaturants on halophilic proteins: protective role of salts

Haloadaptations are perceived to impart stability to halophilic proteins against denaturants. Increasing evidences have gathered now to indicate the role of salt in protecting proteins against denaturants. Studies show that in the presence of salt, secondary and tertiary structure are maintained rigidly against denaturants. However, the question remains the precise mechanism which enables this stability in halophilic enzymes. Few of the important instances have been discussed below.

### Effect of temperature

Thermal stability in proteins is attributed to the combination of factors like improved core packing, increased ionic interactions, decreased hydrophobic surface area, helix stabilization and reduced conformational strain (Sinha and Khare, 2013b). Stability at high temperatures in halophilic proteins has been found to be regulated by presence of salt. β-lactamase from halophilic *Chromohalobacter* sp. retained ~82% of its activity after heat treatment at 100°C for 5 min (Tokunaga et al., 2004). This was indicative of a “reversible renaturation” of the lactamase induced by salt. *Hbt. salinarum* NDK also refolded back post heat treatment in a similar manner at higher concentrations of salt (Ishibashi et al., 2002). “Irreversible aggregation” may have possibly been averted due to the presence of acidic amino acid residues on the protein surface. In another study, higher thermal stability was imparted to *Hbt.* sp. SP1(1) by 4 M NaCl than 2 M (Akolkar and Desai, 2010).

### Effect of chaotropic agents

Investigations on the effect of urea or GdmCl on some halophilic proteins revealed that these are relatively more stable toward denaturation compared to their non-halophilic analog (Dodia et al., 2008; Karan and Khare, 2011). Protective role of salt against urea induced denaturation has been evidenced in case of NDK from *Nab. magadii* (Polosina et al., 2002). At 3.5 M NaCl, it retained complete activity even at 6 M urea. This unique stability was attributed to the possible formation of strong intersubunit contacts within the quaternary structure of the halophilic protein which may have imparted stability to the subdomains and prevented denaturation. Fluorescence spectral analysis established that *Har. hispanica* α-amylase, at 4 M NaCl remained fully folded and conformationally active even in the presence of 6 M urea (Hutcheon et al., 2005). The protein was less structured in absence of salt and gradually lost its overall structure upon increasing urea concentrations. It is likely that charge screening effect imposed by salt ions may have prevented the urea from coming in close vicinity of the polar patches on halophilic protein thereby averting their interaction and subsequent denaturation.

### Effect of organic solvents

Organic solvents behave as mild chaotropic agents. They disrupt hydrogen bonds between protein subunits and reduce the catalytic efficiency by affecting the critical water concentration at the active site. Solvent stability is increasingly being evidenced as a generic trait among halophilic enzymes (Gupta and Khare, 2009). The presence of high salt reduces the water activity significantly. Halophilic enzymes are thus uniquely adapted to function in low water/ non-aqueous media (Kumar and Khare, 2012). Significant solvent stability among halophilic enzymes has been reported by different groups (Shafiei et al., 2011; Li and Yu, 2012; Li et al., 2012; Sinha and Khare, 2013a). However, very little has been investigated about the structure of halophilic enzymes in organic solvents.

Recently, the solvent induced conformational changes have been assessed. Fluorescence investigations of halophilic alcohol dehydrogenase (ADH2) from *Hfx. volcanii* affirmed that salt influences the correct folding of proteins in organic solvents (Alsafadi and Paradisi, 2013). The α-helical content of *Geomicrobium* sp. protease remained unaffected in 50% (v/v) n-hexane and n-decane in presence of 5% (w/v) NaCl (Karan and Khare, 2011). Withdrawal of salt caused loss of α-helical structure.

### Effect of mutations

Preliminary understanding about the mechanism of salt supported structural protection has also emerged from site directed mutagenesis (SDM) experiments. The importance of acidic peptide motif in halophilic enzymes to withstand saline stress was shown by Evilla and Hou (2006). Presence of insertion peptide in extreme halophile *Hbt.* sp. NRC-1 cysteinyl tRNA synthetase (CysRS) showed strong salt dependence, and enhanced enzyme stability at low salt. Deletion of the motif reduced aminoacylation efficiency.

Extensive SDM on halophilic MDH from *Har. marismortui* produced a mutant which was more “halophilic” than the wild type enzyme (Madern et al., 1995). Its structure was solved allowing highlighting the important role of protein solvent interactions in the stabilization of a halophilic protein (Richard et al., 2000). Others SDM studies on Hm MalDH have also demonstrated the important role of anion binding site in the stabilization process (Madern et al., 2000; Irimia et al., 2003; Madern and Ebel, 2007). Mutation of several solvent exposed acidic amino acid residues with lysine in *Hfx. mediterranei* glucose dehydrogenase resulted in proteins displaying a slightly less halophilic behavior than the wild type enzyme (Esclapez et al., 2007). Mutation studies on glucose dehydrogenase and isocitrate dehydrogenase from extremely halophilic Archaea *Hfx. mediterranei* and *Hfx. volcanii* have also recently contributed to understanding of the molecular basis of salt tolerance for halophilic adaptation (Esclapez et al., 2013).

Replacement of 7 Ser residues in thermolysin by Asp improved stability and activity of its mutants (Takita et al., 2008). In the presence of 4 M NaCl, hydrolytic activity of all mutants increased 17–19 folds. Halophilic characteristics were imparted to *Pseudomonas* NDK by replacing 2 Ala residues at its C-terminal end with acidic residues (Glu-Glu) (Tokunaga et al., 2008). Conversely, introduction of Ala-Ala into *Halomonas* sp. 593 NDK (Ha NDK) in place of Glu-Glu caused loss of enzyme halophilicity and critically affected the enzyme properties as well as secondary structure. While the wild type Ha NDK remained stable against dilution induced inactivation, the mutant form was easily irreversibly destabilized by dilution, regardless of presence of salt.

Above studies highlight that protein halophilicity bears a strong correlation with (i) the necessity and importance of acidic amino acid residues on its surface and (ii) the presence of salt which serves important role in restoring/preserving functionality in a halophilic enzyme. On the basis of available data, we may infer that there possibly exists a subset among halophilic proteins which successfully retain structure and activity even under unfavorable conditions. Although, this cannot be generalized, the ability of moderately halophilic enzymes to withstand denaturing environments has not been explored much and provides ample scope for future research.

## Conclusion

The article reviews the present understanding about the responses of halophilic enzymes in solution toward chaotropic reagents and denaturants. Salt is essential in maintaining native structure of halophilic proteins. Sufficient experimental evidences conclude that salt significantly contributes in the modulation of the protein structure/activity toward different chaotropic conditions. The salt induced protective effect on the structure against chaotropic agents, temperature and solvents as well the corresponding structural transitions establish a unique structure-function correlation in halophilic enzymes. Comprehending their differential behavior and stability under harsher conditions could lead to better understanding about the biochemical and biophysical characteristics of these proteins and their exploitation for applications in biocatalysis or biotransformation under saline or low water conditions.

### Conflict of interest statement

The authors declare that the research was conducted in the absence of any commercial or financial relationships that could be construed as a potential conflict of interest.

## References

[B1] Akolkar A. V. Desai A. J. (2010). Catalytic and thermodynamic characterization of protease from *Halobacterium* sp. SP1(1). Res. Microbiol. 161, 355–362 10.1016/j.resmic.2010.04.005 20438836

[B2] Alsafadi D. Paradisi F. (2013). Effect of organic solvents on the activity and stability of halophilic alcohol dehydrogenase (ADH2) from *Haloferax volcanii*. Extremophiles 17, 115–122 10.1007/s00792-012-0498-0 23179592

[B3] Bandyopadhyay A. K. Sonawat H. M. (2000). Salt dependent stability and unfolding of [Fe2-S2] ferredoxin of *Halobacterium salinarum*: spectroscopic investigations. Biophys. J. 79, 501–510 10.1016/S0006-3495(00)76312-0 10866976 PMC1300954

[B4] Bhuyan A. K. (2010). On the mechanism of SDS-induced protein denaturation. Biopolymers 93, 186–199 10.1002/bip.21318 19802818

[B5] Bonneté F. Madern D. Zaccai G. (1994). Stability against denaturation mechanisms in halophilic malate dehydrogenase “adapt” to solvent conditions. J. Mol. Biol. 244, 436–447 10.1006/jmbi.1994.1741 7990132

[B6] Coquelle N. Talon R. Juers D. H. Girard É. Kahn R. Madern D. (2010). Gradual adaptive changes of a protein facing high salt concentrations. J. Mol. Biol. 404, 493–505 10.1016/j.jmb.2010.09.055 20888835

[B7] Costenaro L. Zaccai G. Ebel C. (2002). Link between protein-solvent and weak protein-protein interactions gives insight into halophilic adaptation. Biochemistry 41, 13245–13252 10.1021/bi025830z 12403626

[B8] Danson M. J. Hough D. W. (1997). The structural basis of protein halophilicity. Comp. Biochem. Physiol. A Physiol. 117, 307–312 10.1016/S0300-9629(96)00268-X

[B9] Dodia M. S. Bhimani H. G. Rawal C. M. Joshi R. H. Singh S. P. (2008). Salt dependent resistance against chemical denaturation of alkaline protease from a newly isolated haloalkaliphilic *Bacillus* sp. Bioresour. Technol. 99, 6223–6227 10.1016/j.biortech.2007.12.020 18215518

[B10] Ebel C. Costenaro L. Pascu M. Faou P. Kernel B. Proust-De Martin F. (2002). Solvent interactions of halophilic malate dehydrogenase. Biochemistry 41, 13234–13244 10.1021/bi0258290 12403625

[B11] Ebel C. Faou P. Kernel B. Zaccai G. (1999). Relative role of anions and cations in the stabilization of halophilic malate dehydrogenase. Biochemistry 38, 9039–9047 10.1021/bi9900774 10413477

[B12] Eisenberg H. Mevarech M. Zaccai G. (1992). Biochemical, structural, and molecular genetic aspects of halophilism. Adv. Protein. Chem. 43, 1–62 10.1016/S0065-3233(08)60553-7 1442321

[B13] Esclapez J. Camacho M. Pire C. Bonete M. J. (2013). Biochemical analysis of halophilic dehydrogenases altered by site-directed mutagenesis, in Genetic Manipulation of DNA and Protein - Examples From Current Research, ed D. Figurski (InTech). 10.5772/34565 Available online at: http://cdn.intechopen.com/pdfs/42535/InTech-Biochemical_analysis_of_halophilic_dehydrogenases_altered_by_site_directed_mutagenesis.pdf

[B14] Esclapez J. Pire C. Bautista V. Martínez-Espinosa R. M. Ferrer J. Bonete M. J. (2007). Analysis of acidic surface of *Haloferax mediterranei* glucose dehydrogenase by site-directed mutagenesis. FEBS Lett. 581, 837–842 10.1016/j.febslet.2007.01.054 17289028

[B15] Evilla C. Hou Y. (2006). Acquisition of an insertion peptide for efficient aminoacylation by a halophile tRNA synthetase. Biochemistry 45, 6835–6845 10.1021/bi0521386 16734420

[B16] Ferrer J. Cremades R. Pire C. Bonete M. J. (1998). Fluorescence and quenching comparative studies of halophilic and bovine glutamate dehydrogenase. J. Photochem. Photobiol. B. 47, 148–154 10.1016/S1011-1344(98)00214-0 10093914

[B17] Gafni A. Werber M. M. (1979). Ferredoxin from Halobacterium of the Dead Sea. Structural properties revealed by fluorescence techniques. Arch. Biochem. Biophys. 196, 363–370 10.1016/0003-9861(79)90588-5 485157

[B18] Gupta A. Khare S. K. (2009). Enzymes from solvent tolerant microbes: useful biocatalysts for non-aqueous enzymology. Crit. Rev. Biotechnol. 29, 44–54 10.1080/07388550802688797 19514902

[B19] Hutcheon G. W. Vasisht N. Bolhuis A. (2005). Characterisation of a highly stable α-amylase from the halophilic archaeon *Haloarcula hispanica*. Extremophiles 9, 487–495 10.1007/s00792-005-0471-2 16075161

[B20] Inouye K. Kuzuya K. Tonomura B. (1998). Sodium chloride enhances markedly the thermal stability of thermolysin as well as its catalytic activity. Biochim. Biophys. Acta 1388, 209–214 10.1016/S0167-4838(98)00189-7 9774734

[B21] Irimia A. Ebel C. Madern D. Richard S. B. Cosenza L. W. Zaccaï G. (2003). The oligomeric states of *Haloarcula marismortui* malate dehydrogenase are modulated by solvent components as shown by crystallographic and biochemical studies. J. Mol. Biol. 326, 859–873 10.1016/S0022-2836(02)01450-X 12581646

[B22] Ishibashi M. Arakawa T. Philo J. S. Sakashita K. Yonezawa Y. Tokunaga H. (2002). Secondary and quaternary structural transition of the halophilic archaeon nucleoside diphosphate kinase under high- and low-salt conditions. FEMS Microbiol. Lett. 216, 235–241 10.1111/j.1574-6968.2002.tb11441.x 12435508

[B23] Karan R. Khare S. K. (2011). Stability of haloalkaliphilic *Geomicrobium* sp. protease modulated by salt. Biochemistry (Mosc.) 76, 686–693 10.1134/S0006297911060095 21639849

[B24] Kumar S. Khare S. K. (2012). Purification and characterization of maltooligosaccharide-forming alpha-amylase from moderately halophilic *Marinobacter* sp. EMB8. Bioresour. Technol. 116, 247–251 10.1016/j.biortech.2011.11.109 22197336

[B25] Lanyi J. K. (1974). Salt-dependent properties of proteins from extremely halophilic bacteria. Bacteriol. Rev. 38, 272–290 4607500 10.1128/br.38.3.272-290.1974 PMC413857

[B26] Larsen R. W. Yang J. Hou S. Helms M. K. Jameson D. M. Alam M. (1999). Spectroscopic characterization of two soluble transducers from the archaeon *Halobacterium salinarum*. J. Protein Chem. 18, 269–275 10.1023/A:1021031227197 10395445

[B27] Li X. Wang H. Li T. Yu H. (2012). Purification and characterization of an organic solvent-tolerant alkaline cellulase from a halophilic isolate of Thalassobacillus. Biotechnol. Lett. 34, 1531–1536 10.1007/s10529-012-0938-z 22538547

[B28] Li X. Yu H. Y. (2012). Purification and characterization of novel organic-solvent-tolerant β-amylase and serine protease from a newly isolated *Salimicrobium halophilum* strain LY20. FEMS Microbiol. Lett. 329, 204–211 10.1111/j.1574-6968.2012.02522.x 22324975

[B29] Lindgren M. Westlund P. O. (2010). On the stability of chymotrypsin inhibitor 2 in a 10 M urea solution. The role of interaction energies for urea-induced protein denaturation. Phys. Chem. Chem. Phys. 12, 9358–9366 10.1039/b925726h 20563326

[B30] Madern D. Camacho M. Rodríguez-Arnedo A. Bonete M. J. Zaccai G. (2004). Salt-dependent studies of NADP-dependent isocitrate dehydrogenase from the halophilic archaeon *Haloferax volcanii*. Extremophiles 8, 377–384 10.1007/s00792-004-0398-z 15221656

[B31] Madern D. Ebel C. (2007). Influence of an anion-binding site in the stabilization of halophilic malate dehydrogenase from *Haloarcula marismortui*. Biochimie 89, 981–987 10.1016/j.biochi.2007.03.008 17451860

[B32] Madern D. Ebel C. Zaccai G. (2000). Halophilic adaptation of enzymes. Extremophiles 4, 91–98 10.1007/s007920050142 10805563

[B33] Madern D. Pfister C. Zaccai G. (1995). Mutation at a single acidic amino acid enhances the halophilic behaviour of malate dehydrogenase from *Haloarcula marismortui* in physiological salts. Eur. J. Biochem. 230, 1088–1095 10.1111/j.1432-1033.1995.tb20659.x 7601139

[B34] Madern D. Zaccai G. (1997). Stabilisation of halophilic malate dehydrogenase from *Haloarcula marismortui* by divalent cations. Eur. J. Biochem. 249, 607–611 10.1111/j.1432-1033.1997.00607.x 9370373

[B35] Madern D. Zaccai G. (2004). Molecular adaptation: the malate dehydrogenase from the extreme halophilic bacterium *Salinibacter ruber* behaves like a non-halophilic protein. Biochimie 86, 295–303 10.1016/j.biochi.2004.04.004 15194233

[B36] Mevarech M. Eisenberg H. Neumann E. (1977). Malate dehydrogenase isolated from extremely halophilic bacteria of the Dead Sea. 1. Purification and molecular characteristics. Biochemistry 16, 3781–3785 10.1021/bi00636a009 901751

[B37] Mevarech M. Frolow F. Gloss L. M. (2000). Halophilic enzymes: proteins with a grain of salt. Biophys. Chem. 86, 155–164 10.1016/S0301-4622(00)00126-5 11026680

[B38] Müller-Santos M. de Souza E. M. Pedrosa F. O. Mitchell D. A. Longhi S. Carrière F. (2009). First evidence for the salt-dependent folding and activity of an esterase from the halophilic archaea *Haloarcula marismortui*. Biochim. Biophys. Acta 1791, 719–729 10.1016/j.bbalip.2009.03.006 19303051

[B39] Oren A. (2008). Microbial life at high salt concentrations: phylogenetic and metabolic diversity. Saline Syst. 4, 2 10.1186/1746-1448-4-2 18412960 PMC2329653

[B40] Oren A. (2013). Life at high salt concentrations, intracellular KCl concentrations, and acidic proteomes. Front. Microbiol. 4:315 10.3389/fmicb.2013.00315 24204364 PMC3817357

[B41] Polosina Y. Y. Zamyatkin D. F. Kostyukova A. S. Filimonov V. V. Fedorov O. V. (2002). Stability of *Natrialba magadii* NDP kinase? comparisons with other halophilic proteins. Extremophiles 6, 135–142 10.1007/s007920100232 12013434

[B42] Pundak S. Eisenberg H. (1981). Structure and activity of malate dehydrogenase of the extreme halophilic bacteria of the Dead Sea. 1. Conformation and interaction with water and salt between 5 M and 1 M NaCl concentration. Eur. J. Biochem. 118, 463–470 10.1111/j.1432-1033.1981.tb05542.x 7297556

[B43] Rao L. Zhao X. Pan F. Li Y. Xue Y. Ma Y. (2009). Solution behavior and activity of a halophilic esterase under high salt concentration. PLoS ONE 4:e6980 10.1371/journal.pone.0006980 19759821 PMC2736375

[B44] Richard S. B. Madern D. Garcin E. Zaccai G. (2000). Halophilic adaptation: novel solvent protein interactions observed in the 2.9 and 2.6 Å resolution structures of the wild type and a mutant of malate dehydrogenase from *Haloarcula marismortui*. Biochemistry 39, 992–1000 10.1021/bi991001a 10653643

[B45] Shafiei M. Ziaee A. A. Amoozegar M. A. (2011). Purification and characterization of an organic-solvent-tolerant halophilic α-amylase from the moderately halophilic *Nesterenkonia* sp. strain F. J. Ind. Microbiol. Biotechnol. 38, 275–281 10.1007/s10295-010-0770-1 20593298

[B46] Sinha R. Khare S. K. (2013a). Characterization of detergent compatible protease of a halophilic *Bacillus* sp. EMB9?: differential role of metal ions in stability and activity. Bioresour. Technol. 145, 357–361 10.1016/j.biortech.2012.11.024 23219691

[B47] Sinha R. Khare S. K. (2013b). Thermostable proteases, in Thermophilic Microbes in Environmental and Industrial Biotechnology, eds Satyanarayana T. Littlechild J. Kawarabayasi Y. (Dordrecht: Springer), 859–880 10.1007/978-94-007-5899-5_32

[B48] Souza T. A. Okamoto D. N. Ruiz D. M. Oliveira L. C. Kondo M. Y. Tersario I. L. (2012). Correlation between catalysis and tertiary structure arrangement in an archaeal halophilic subtilase. Biochimie 94, 798–805 10.1016/j.biochi.2011.11.011 22177966

[B49] Tadeo X. López-Méndez B. Trigueros T. Laín A. Castaño D. Millet O. (2009). Structural basis for the aminoacid composition of proteins from halophilic archea. PLoS Biol. 7:e1000257 10.1371/journal.pbio.1000257 20016684 PMC2780699

[B50] Takita T. Aono T. Sakurama H. Itoh T. Wada T. Minoda M. (2008). Effects of introducing negative charges into the molecular surface of thermolysin by site-directed mutagenesis on its activity and stability. Biochim. Biophys. Acta 1784, 481–488 10.1016/j.bbapap.2007.12.004 18187054

[B51] Talon R. Coquelle N. Madern D. Girard E. (2014). An eperimental point of view on hydration/ solvation in halophilic proteins. Front. Microbiol. 5:66 10.3389/fmicb.2014.00066 24600446 PMC3930881

[B52] Tannous E. Yokoyama K. You D. J. Koga Y. Kanaya S. (2012). A dual role of divalent metal ions in catalysis and folding of RNase H1 from extreme halophilic archaeon *Halobacterium* sp. NRC-1. FEBS Open Bio. 2, 345–352 10.1016/j.fob.2012.10.003 23772368 PMC3678122

[B53] Tokunaga H. Arakwa T. Tokunaga M. (2008). Engineering of halophilic enzymes: two acidic amino acid residues at the carboxy-terminal region confer halophilic characteristics to *Halomonas* and *Pseudomona*s nucleoside diphosphate kinases. Protein Sci. 17, 1603–1610 10.1110/ps.035725.108 18573868 PMC2525524

[B54] Tokunaga H. Ishibashi M. Arakawa T. Tokunaga M. (2004). Highly efficient renaturation of β-lactamase isolated from moderately halophilic bacteria. FEBS Lett. 558, 7–12 10.1016/S0014-5793(03)01508-4 14759507

[B55] Wright D. B. Banks D. D. Lohman J. R. Hilsenbeck J. L. Gloss L. M. (2002). The effect of salts on the activity and stability of *Escherichia coli* and *Haloferax volcanii* dihydrofolate reductases. J. Mol. Biol. 323, 327–344 10.1016/S0022-2836(02)00916-6 12381324

[B56] Zaccai G. (2013). Hydration shells with a pinch of salt. Biopolymers 99, 233–238 10.1002/bip.22154 23348670

